# Investigation of the first reported outbreak of New Delhi metallo-β-lactamase-1-producing *Pseudomonas aeruginosa* in Texas

**DOI:** 10.1017/ash.2023.521

**Published:** 2024-01-02

**Authors:** Madhuri M. Sopirala, Kathleen Hartless, Sherry Reid, Angela Christie-Smith, Jeanette Fiveash, Aderonke Badejogbin, Andrew Otto Psenicka

**Affiliations:** 1 Division of Infectious Diseases and Geographic Medicine, Department of Internal Medicine, University of Texas Southwestern Medical Center, Dallas, TX, USA; 2 Infection Prevention and Control Program, VA North Texas Health Care System, Dallas, TX, USA

## Abstract

We describe an epidemiologic investigation and successful control measures for the first reported outbreak of bla_NDM-1_-carrying *Pseudomonas aeruginosa* in Texas occurring in a veteran with transmission of the same organism and a bla_NDM-5_-carrying *Escherichia coli*, respectively, to two roommates and bla_NDM_-carrying organism/s to a patient cared for by common staff.

## Introduction

Carbapenemase-producing organisms are epidemiologically significant due to easily transferred mobile genetic elements such as plasmids and transposons.^1^ Carbapenemases have been responsible for rapid global spread of carbapenem-resistant Enterobacterales.^2^ New Delhi Metallo-Beta-Lactamase (bla_NDM_) is one such carbapenemase first described in 2009 in India.^3^ At the time of this outbreak, Enterobacterales carrying bla_NDM_ had been reported in 34 states of the United States with only 7 reported cases of bla_NDM_-carrying *Pseudomonas aeruginosa* from four states but none from Texas.^4^ We describe a clinical and molecular epidemiologic investigation of the first reported outbreak of bla_NDM_-carrying *P. aeruginosa* in the State of Texas and describe the control measures that were effective in quickly containing the spread of bla_NDM_-carrying organisms at the spinal cord injury center (SCI) and the acute care hospital (ACH) within Veterans Affairs North Texas Health Care System. We also report transmission of bla_NDM-1_-carrying *P. aeruginosa* to one roommate and bla_NDM-5_-carrying *Escherichia coli* to another roommate of the same index patient.

## Methods

### Setting

Veterans Affairs North Texas Health Care System (VANTHCS) serves veterans from 38 counties in Texas and 2 counties in Southern Oklahoma. It has 835 operating beds including an academic ACH and a SCI with 30 beds.

### Investigation period

This outbreak investigation occurred from July 2018 to October 2018.

### Clinical and molecular epidemiologic investigation

A case was defined as any patient admitted to the SCI or the ACH in whom a bla_NDM_-carrying organism was either cultured clinically or a rectal screen with a real-time polymerase chain reaction (PCR) assay (Xpert® Carba-R) was positive for bla_NDM_. The PCR assay from rectal screen is only able to identify the presence and type of the carbapenemase gene but unable to specify the genera of bacteria carrying the gene. An indirect contact was defined as a patient who was cared for by hospital staff who also cared for the index patient. A clinical epidemiologic investigation was launched when the index case was identified with a urine culture positive for bla_NDM_-carrying *P. aeruginosa.* Universal contact isolation was initiated in SCI and all non-critical shared equipment on SCI and ACH unit underwent supervised disinfection. A systematic approach was undertaken with SCI roommates screened initially with a rectal swab for real-time PCR testing to identify any of the five carbapenemase genes including bla_NDM_ expanding investigation to patients outside the index patient’s room once the screen was positive. We reviewed electronic medical record and staffing schedules to identify patients who shared healthcare staff with the index patient in the ACH and approached those patients for consent to screen. We performed phased point prevalence testing with rectal screening three times, four weeks apart. Detection of carbapenemase production and molecular characterization of bla_NDM_ gene using PCR assay were performed as described earlier.^5^ Whole genome sequencing (WGS) was performed by the Centers for Disease Control and Prevention (CDC) with short-read sequencing on all three (Illumina MiSeq) and long-read sequencing on one *Pseudomonas* isolate (PacBio). The project was deemed as quality improvement by an institutional review process and the need for research approval was waived.

## Results

**Figure 1.**
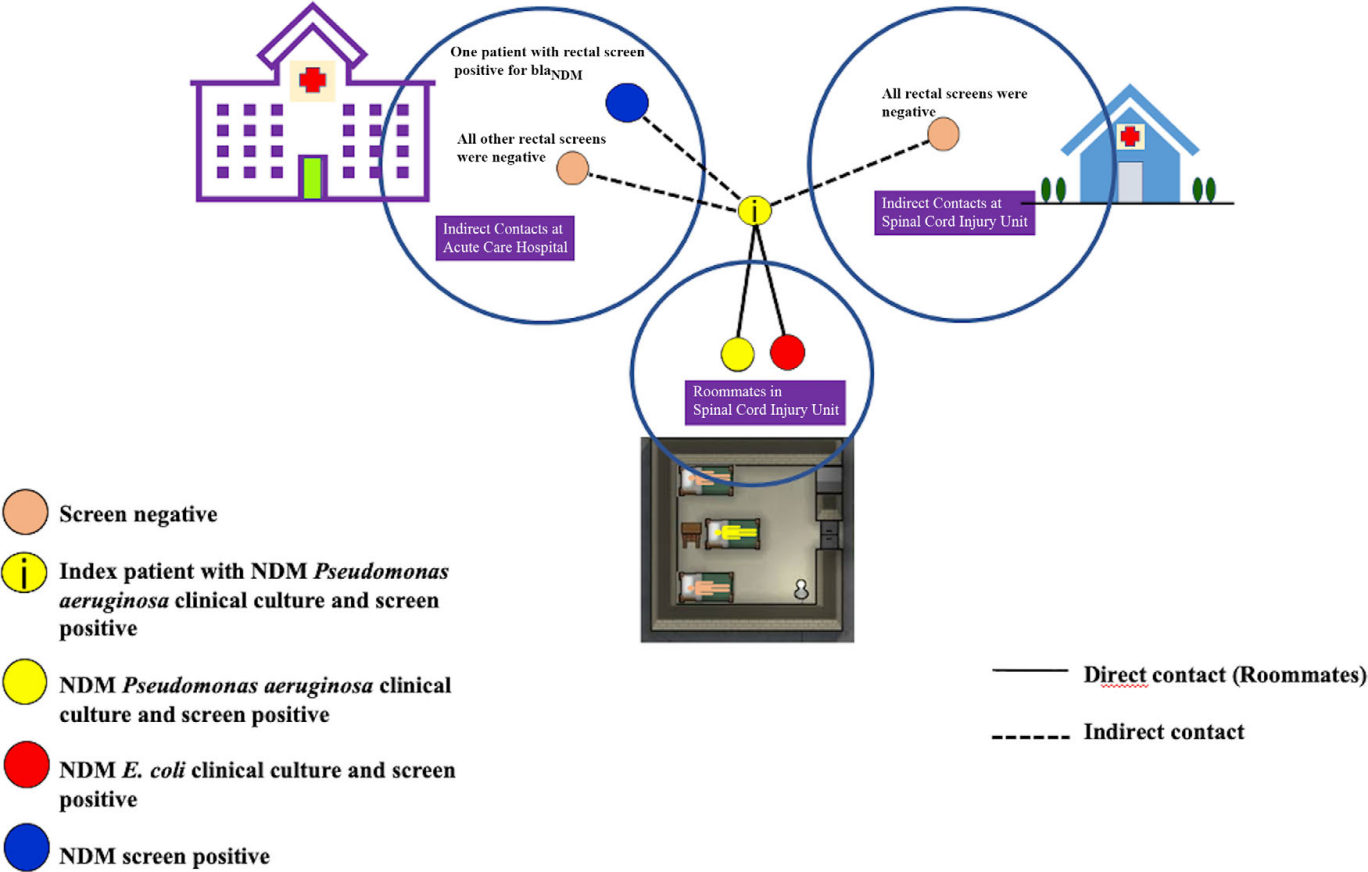
Diagrammatic representation of cases showing bla_NDM_-carrying organisms in indirect and direct contact with the index patient. NDM, New Delhi Metallo-Beta-Lactamase.

Our investigation revealed that the index patient was transferred to the SCI from Thailand following a three-month hospitalization due to injuries sustained from a motor vehicle accident while vacationing overseas. He was transferred the day after admission to the ACH within the same campus due to hypokalemia where he stayed for 7 days and then transferred to SCI sharing a room with two other patients for 31 days before a urine culture grew bla_NDM_-carrying *P. aeruginosa*. Rectal screens performed immediately after this were positive for bla_NDM_ in the index patient and one of the two roommates. Twenty-nine days later, the roommate with positive screen had a urine and a coccyx culture positive for bla_NDM_-carrying *P. aeruginosa,* and the second roommate had a urine culture positive for bla_NDM_-carrying *E. coli*. Treatment was unnecessary during this outbreak as positive cultures were deemed secondary to colonization. A third patient who was an indirect contact in the same unit as the index patient in ACH tested positive for bla_NDM_ with rectal screening (Figure 1). A total of 54 patients were identified as indirect contacts to the index patient. Of the 54 patients, 28 patients underwent rectal screening, the remaining either refused or were unable to get tested. In addition, point prevalence rectal screening was conducted in three phases in the SCI and included a total of 30 patients. All these tests were negative. Whole genome sequencing revealed that index patient and roommate 1 had bla_NDM-1_-carrying *P. aeruginosa* whereas roommate 2 had bla_NDM-5_-carrying *E. coli*. Whole genome sequencing did not reveal any plasmids in bla_NDM-1_-carrying *P. aeruginosa*. No further spread occurred. Our investigation ended after twelve weeks with all the rectal screens testing negative for bla_NDM_ during the three phases of point prevalence testing. Risk factors and comorbidities, time between exposure to positive test, clinical outcome, and resistance genes for all patients identified to be carrying the bla_NDM_ are outlined in Table 1.

**Table 1.** Clinical epidemiologic data and whole genome sequencing in case patients identified to have bla_NDM_

Patient	Days since exposure to index patient to positive culture	Risk factors and comorbidities	Organism	Culture site	Clinical Outcome	Whole genome sequencing Antibiotic resistance genes
1 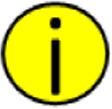	NA	Prior hospitalization, prior antibiotic use, intensive care unit stay, indwelling urinary catheter, wound care, and peripherally inserted central venous catheter	*Pseudomonas aeruginosa*	Urine	Died 98 days after diagnosis; sepsis/urinary tract infection	*bla*_*NDM-1*_ *, aac(6')-ib3, aada1-pm, aada12, aada24, ant(2'')-ia, ant(4')-iib, aph(3'')-ib, aph(3')-iib, aph(6)-id, arr-2, arr-3, bcr1, bla*_*oxa-10*_ *, bla*_*oxa-488*_ *, blapao, blaveb-2, catb7, cml, cmla5, crpp, dfrb2, fosa, sul1, tet(g), tetr(g)*
2 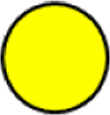	35 days	Prior hospitalization, prior antibiotic use, intensive care unit stay, indwelling urinary catheter, wound care, diabetes mellitus, and post-surgery	*P. aeruginosa*	Urine and coccyx	Died 193 days after diagnosis at an outside hospital; complications due to infection	*bla*_*NDM-1*_ *, aac(6')-ib-hangzhou, aada1-pm, aada24, ant(2'')-ia, ant(4')-iib, aph(3'')-ib, aph(3')-iib, aph(6)-id, arr-2, arr-3, bcr1, bla*_*oxa-10*_ *, bla*_*oxa-488*_ *, bla*_*pao*_ *, bla*_*veb-2*_ *, catb7, cml, cmla5, crpp, dfrb2, fosa, sul1, tet(g), tetr(g)*
3 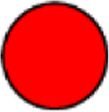	18 days	Prior hospitalization, prior antibiotic use, indwelling urinary catheter, percutaneous endoscopic gastrostomy tube, diabetes mellitus, and post-surgery	*Escherichia coli*	Urine	Alive	bla_NDM-5_, aac(3)-iid, aac(6')-ib-cr, aada2, aada5, ampc1, ampc1_ecoli, ampc2, amph, amph_ecoli, aph(3'')-ib, aph(6)-id, blacmy-2, blactx-m-15, blaoxa-1, blatem-1b, catb4, dfra12, dfra17, mdf(a), mph(a), sul1, sul2, tet(b), Col(BS512), IncFIA, IncFIB(AP001918), IncFII (pAMA1167-NDM-5), IncI2, IncQ1, IncY
4 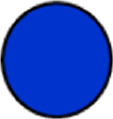	4 days	Prior hospitalization, prior antibiotic use, nursing home stay in the past 3 months, hemodialysis, tunneled catheter, indwelling urinary catheter, congestive heart failure, cerebrovascular accident, diabetes mellitus, and total parenteral nutrition	NA	NA	Transferred to hospice care and died the day after positive screen due to unrelated illness	NA

NA, not applicable; NDM, New Delhi Metallo-Beta-Lactamase.

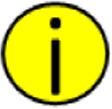
 Index patient with NDM-1 *Pseudomonas aeruginosa* clinical culture and screen positive. 
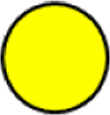
 NDM-1 *Pseudomonas aeruginosa* clinical culture and screen positive. 
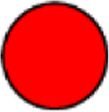
 NDM-5 *E. coli* clinical culture and screen positive. 
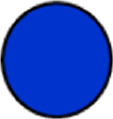
 NDM screen positive with real-time PCR; no culture positive.

## Discussion

*P. aeruginosa* carrying bla_NDM_ has been infrequently detected within the US. However rapid spread of carbapenemase-producing organisms has occurred globally likely owing to international travel followed by local spread. For example, the first KPC-producing *Klebsiella pneumoniae* was discovered in the United States in 1996 from a hospital in North Carolina.^6^ Since then, there has been exponential growth in KPC prevalence in the US (CDC, https://arpsp.cdc.gov/profile/antibiotic-resistance). In addition to sporadic international travel-related cases, occasional domestically acquired NDM *P. aeruginosa* have been reported.^7^ Our efforts to control the bla_NDM_-carrying organisms have quickly mitigated further spread within our SCI and ACH.

One of the roommates of the index patient had a urine culture positive for *E. coli* with bla_NDM-5_. There was no plasmid detected to indicate intergenus transfer of *bla*_NDM_. It is very likely that the index patient carried both bla_NDM-1_-carrying *P. aeruginosa* and bla_NDM-5_-carrying *E. coli* and transmitted one each to each of his two roommates. bla_NDM-5_ was first recovered in the United Kingdom in a patient with a history of travel to the Indian subcontinent.^8^

Our study has its limitations. First, this is a single-centered, observational study in a specific patient population, which may not be generalizable to other care settings. Nevertheless, we demonstrated that timely interventions helped us limit the outbreak to a small number of patients.^9,10^ By promptly identifying and isolating direct and indirect contacts, we were able to limit the transmission to the three contact patients with two of them being index patient’s roommates. Second, we only screened indirect contacts whom we defined as those that had common healthcare staff with the index patient. We did not explore colonization in patients who may have shared non-critical medical equipment and therefore cannot ensure that there was no transmission to the patients who were not screened. However, we have not seen any subsequent infections develop in any of our veterans. In conclusion, timely interventions including prompt isolation of colonized patients were effective in curbing transmission of bla_NDM_-carrying organisms. It is very likely that our index patient was colonized with both bla_NDM-1_-carrying *P. aeruginosa* and bla_NDM-5_-carrying *E. coli* and transferred one each to each of his roommates; in addition, transferred bla_NDM_-carrying organism/s to another patient who is an indirect contact, either through common staff or shared equipment.
